# Aging‐related carcinoembryonic antigen‐related cell adhesion molecule 1 signaling promotes vascular dysfunction

**DOI:** 10.1111/acel.13025

**Published:** 2019-08-06

**Authors:** Florian Kleefeldt, Heike Bömmel, Britta Broede, Michael Thomsen, Verena Pfeiffer, Philipp Wörsdörfer, Srikanth Karnati, Nicole Wagner, Uwe Rueckschloss, Süleyman Ergün

**Affiliations:** ^1^ Institute of Anatomy and Cell Biology Julius‐Maximilians‐University Würzburg Würzburg Germany; ^2^ Leonardo Hirslanden Clinic Birshof Münchenstein Switzerland; ^3^ Albertinen Hospital, Cardiac Anesthesia Hamburg Germany

**Keywords:** aging, anti‐aging, cytokines, inflammation, mouse, reactive oxygen species

## Abstract

Aging is an independent risk factor for cardiovascular diseases and therefore of particular interest for the prevention of cardiovascular events. However, the mechanisms underlying vascular aging are not well understood. Since carcinoembryonic antigen‐related cell adhesion molecule 1 (CEACAM1) is crucially involved in vascular homeostasis, we sought to identify the role of CEACAM1 in vascular aging. Using human internal thoracic artery and murine aorta, we show that CEACAM1 is upregulated in the course of vascular aging. Further analyses demonstrated that TNF‐α is CEACAM1‐dependently upregulated in the aging vasculature. Vice versa, TNF‐α induces CEACAM1 expression. This results in a feed‐forward loop in the aging vasculature that maintains a chronic pro‐inflammatory milieu. Furthermore, we demonstrate that age‐associated vascular alterations, that is, increased oxidative stress and vascular fibrosis, due to increased medial collagen deposition crucially depend on the presence of CEACAM1. Additionally, age‐dependent upregulation of vascular CEACAM1 expression contributes to endothelial barrier impairment, putatively via increased VEGF/VEGFR‐2 signaling. Consequently, aging‐related upregulation of vascular CEACAM1 expression results in endothelial dysfunction that may promote atherosclerotic plaque formation in the presence of additional risk factors. Our data suggest that CEACAM1 might represent an attractive target in order to delay physiological aging and therefore the transition to vascular disorders such as atherosclerosis.

## 1 INTRODUCTION

The English physician Thomas Sydenham (1624–1689) once noted that “a man is as old as his arteries” (Ungvari, 2015). Today, we know that age is an independent risk factor for atherosclerosis as well as secondary diseases such as myocardial infarction and stroke (Wang & Bennett, 2012) that represent the main cause of death worldwide (Gisterå & Hansson, 2017). Therefore, understanding the processes of vascular aging is of greatest significance to prevent the onset of cardiovascular diseases. However, compared to pathological conditions such as atherosclerosis, physiological vascular aging is less well understood (Xu et al., 2017).

On the macroscopic level, aging alters the mechanical properties of blood vessels by increasing vascular stiffness. This is due to a reduced content of elastic fibers, but increased vascular collagen deposition, calcification, and intimal thickening (Collins, Munoz, Patel, Loukas & Tubbs, 2014; Tonar et al., 2015). Intimal thickening is present in older people even in populations with low incidence of atherosclerosis (Virmani et al., 1991) and predicts cardiovascular events (Okayama et al., 2013).

Besides these macroscopic alterations, aging is associated with a shift toward a pro‐inflammatory milieu. This is illustrated by the finding that the level of TNF‐α, a very important pro‐inflammatory mediator, is elevated in serum as well as in the vasculature of aging humans and rodents (Csiszar, Ungvari, Koller, Edwards & Kaley, 2003; Paolisso et al., 1998; Spaulding, Walford & Effros, 1997). Age‐dependent inflammation is associated with increased vascular oxidative stress and contributes to the development of endothelial dysfunction (ED) (Rodríguez‐Mañas et al., 2009). Since ED may initiate vascular disorders such as atherosclerosis (Bömmel et al., 2017; Dantas, Jiménez‐Altayó & Vila, 2012; Rodríguez‐Mañas et al., 2009), ED links physiological aging to the onset of pathological processes. Furthermore, ED predicts cardiovascular events in humans (Gokce et al., 2003; Gutiérrez et al., 2013; Hadi, Carr & Al Suwaidi, 2005). Therefore, elucidating the mechanisms of aging‐associated ED development in more detail will help to prevent cardiovascular secondary diseases such as myocardial infarction and stroke in the future.

Enhanced vascular permeability is a hallmark of ED (Bömmel et al., 2017; Ichiki et al., 2013; Phinikaridou et al., 2012). The carcinoembryonic antigen‐related cell adhesion molecule 1 (CEACAM1), a member of the immunoglobulin superfamily, is known to regulate vascular permeability (Nouvion et al., 2010). On the one hand, CEACAM1 is an important effector of VEGF/VEGFR‐2 signaling (Ergün et al., 2000) that is one of the most potent inducers of vascular hyperpermeability (Bates, 2010). On the other hand, CEACAM1 was shown to upregulate the endothelial expression of VEGF and VEGFR‐2 (Kilic et al., 2005). Furthermore, we have previously shown that in the presence of TNF‐α, CEACAM1 recruits Src kinase to intercellular adhesion sites, thereby promoting the phosphorylation of adherens junction proteins and increasing vascular permeability (Ghavampour et al., 2018).

Based on these findings, we hypothesized that CEACAM1 could be involved in age‐associated ED development. However, a role for CEACAM1 in vascular aging has not been proven so far. Therefore, the aim of the present study was to elucidate the impact of CEACAM1 on physiological vascular aging with respect to the initiation of age‐dependent ED by analysis of human and murine vascular specimens.

## 2 RESULTS

### 2.1 Lack of pathological alterations identify HITA as a suitable model of physiological vascular aging

**Figure 1:**
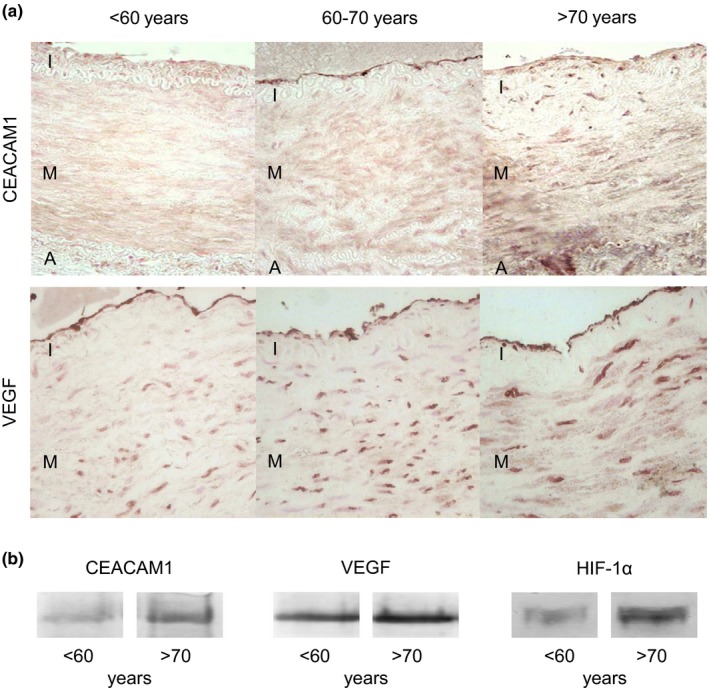
Expression of carcinoembryonic antigen‐related cell adhesion molecule 1 (CEACAM1) and VEGF is upregulated in aging human vasculature. (a) Human internal thoracic artery (HITA) specimens were grouped according to age in three groups: <60, 60–70 and >70 years. Immunohistochemical analyses demonstrated an age‐dependent upregulation of CEACAM1 and VEGF, especially within the intima. (b) Similar results were obtained by immunoblotting. The transcription factor HIF‐1α that is involved in VEGF gene expression was also upregulated with advanced age. A, adventitia; I, intima; M, media

Extensive morphological analyses revealed that human internal thoracic artery (HITA) specimens display hardly any pathological alterations such as atherosclerosis, intimal thickening, or inflammatory processes. Infiltration of inflammatory cells was detected only in one HITA sample (data not shown). Comparison of HITA specimens obtained from patients of different age (Table S1) demonstrated that normal vascular morphology was maintained even in advanced age (Figure S1a–d). In some HITA specimens, a slight intimal thickening was observed (Figure 1e,f). However, more than 90% of HITA specimens did not show any vascular alterations. In comparison, 66% of radial artery specimens displayed vascular alterations such as intimal thickening or atherosclerotic plaque development (Figure 1g,h). Therefore, HITA represents a suitable model to analyze physiological vascular aging.

### 2.2 Aging increases expression of CEACAM1, VEGF, and HIF‐1α in HITA

Carcinoembryonic antigen‐related cell adhesion molecule 1 regulates endothelial barrier function in an age‐dependent manner (Ghavampour et al., 2018). To address the question whether CEACAM1 expression is altered in the course of physiological aging, HITA specimens grouped according to the donor's age (<60, 60–70, and >70 years) were analyzed immunohistochemically (Figure 1a). These analyses showed a distinct upregulation of CEACAM1 expression within the intima of patients aged 60–70 and >70 years. In contrast, CEACAM1 was hardly detectable in HITA specimens from patients younger than 60 years. Besides the intima, a slight CEACAM1 staining could be detected within the media that moderately increased with age (Figure 1a).

Carcinoembryonic antigen‐related cell adhesion molecule 1 is known to influence VEGF/VEGFR‐2 signaling (Ergün et al., 2004). According to the results obtained for CEACAM1, immunohistochemical analyses of HITA specimens revealed a concomitant increase in VEGF expression with progressive age, especially within the intima and media of the vascular wall (Figure 1a). Besides these findings, a distinct expression of CEACAM1 was abundant in vasa vasorum only in patients older than 60 years (Figure 1i,j). Immunoblot analyses confirmed the age‐dependent upregulation of CEACAM1 and VEGF (Figure 1b).

Since HIF‐1α induces expression of VEGF (Chen, Endler & Shibasaki, 2009), HIF‐1α expression was analyzed by immunoblotting. Similar to VEGF, HIF‐1α is upregulated in HITA specimens from older patients (Figure 1b).

### 2.3 Aging increases expression of CEACAM1, VEGF, and VEGFR‐2 in murine aorta

In order to dissect the role of CEACAM1 in vascular aging, we used a murine model of genetic ablation of CEACAM1, the *Cc1* ^*−/−*^ mouse (Leung et al., 2006; Najjar et al., 2013).

**Figure 2:**
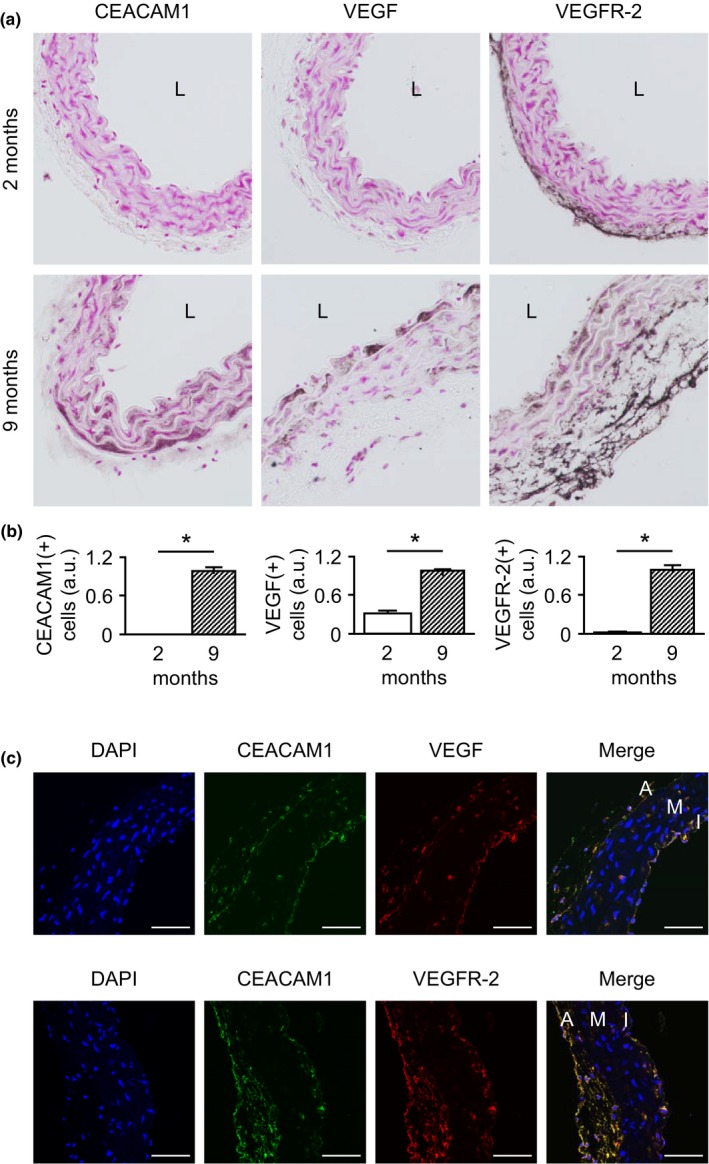
Expression of carcinoembryonic antigen‐related cell adhesion molecule 1 (CEACAM1), VEGF, and VEGFR‐2 is age‐dependently upregulated in murine aortae. (a) Immunohistochemical staining of aortic cross sections derived from 2‐ to 9‐month‐old mice. Aortae of 9‐month‐old mice showed a distinct upregulation of CEACAM1, VEGF, and VEGFR‐2 compared to aortae of 2‐month‐old mice. (b) Quantification of CEACAM1(+), VEGF(+), and VEGFR‐2(+) cells within cross sections of 2‐ and 9‐month‐old mice. Cell count was normalized to the inner aortic circumference. For all three proteins, the number of positive cells increased with age. (c) Immunohistochemical analyses of aortic cross sections of 9‐month‐old mice. Especially within the intima and adventitia, a high number of cells co‐expressing CEACAM1 with VEGF and VEGFR‐2, respectively, were present. A, adventitia; I, intima; L, lumen; M, media. *p < 0.05

We first sought to verify our results obtained from HITA analyses in the murine model using animals aged 2–3 and 9 months, respectively. Similar to HITA, the number of CEACAM1(+) cells and VEGF(+) cells within the aorta increased in 9‐month‐old WT mice compared to 2‐month‐old mice. In the case of CEACAM1, aortic expression was undetectable in younger mice. Furthermore, we analyzed the presence of VEGFR‐2, the receptor that predominantly mediates most endothelial VEGF effects. Similar to VEGF, 9‐month‐old WT mice showed a tremendous increase in VEGFR‐2(+) cells within the intima (Figure 2a,b). Altogether, we found that the age‐dependent alterations in the expression of CEACAM1 and VEGF observed in HITA specimens are similarly detectable in the murine model. Therefore, we conclude that the murine model is suitable to investigate the contribution of CEACAM1 to physiological vascular aging.

### 2.4 CEACAM1 is involved in age‐dependent aortic expression of VEGF and VEGFR‐2

Since CEACAM1, VEGF, and VEGFR‐2 showed a similar distribution pattern in the aging vasculature, we conducted co‐immunohistochemistry for CEACAM1 and VEGF or VEGFR‐2 in aortae of 9‐month‐old mice. These analyses demonstrated a great number of cells co‐expressing CEACAM1 with VEGF as well as CEACAM1 with VEGFR‐2, especially within the intima (Figure 2c).

**Figure 3:**
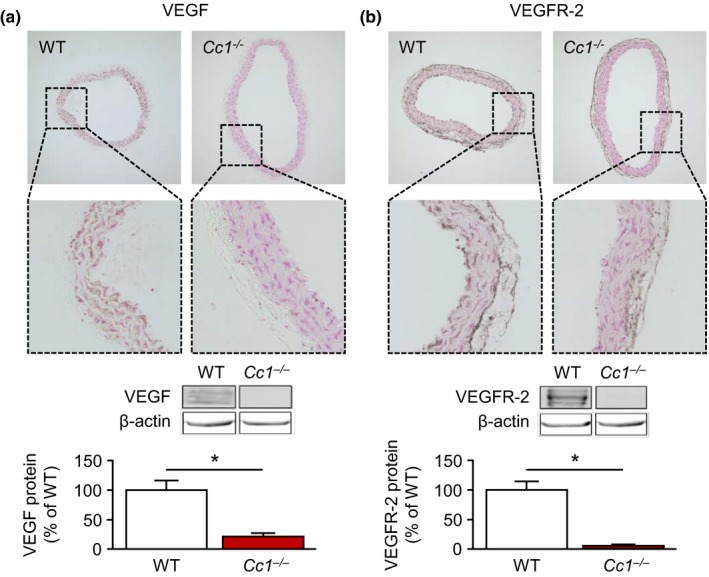
Carcinoembryonic antigen‐related cell adhesion molecule 1 (CEACAM1) promotes aortic expression of VEGF and VEGFR‐2 in 9‐month‐old mice. (a) CEACAM1 deficiency resulted in a reduced expression of VEGF as indicated by immunohistochemical analyses of murine aortic cross sections and immunoblotting, respectively. (b) Similar to VEGF, expression of VEGFR‐2 was lower in aortae of *Cc1* ^*−/−*^ compared to WT mice at the age of 9 months. *p < 0.05

In order to analyze the contribution of CEACAM1 to the age‐dependent alterations in aortic VEGF and VEGFR‐2 expression, aortae of 9‐month‐old WT and *Cc1* ^*−/−*^ mice were analyzed by immunohistochemistry and immunoblotting. Using both methods, we found that aortic expression of VEGF is higher in WT mice compared to *Cc1* ^*−/−*^ mice (Figure 3a). Similarly, VEGFR‐2 expression was also higher in aortae of aged WT mice (Figure 3b).

### 2.5 CEACAM1 contributes to age‐associated TNF‐α expression, oxidative stress, and stabilization of HIF‐1α

**Figure 4:**
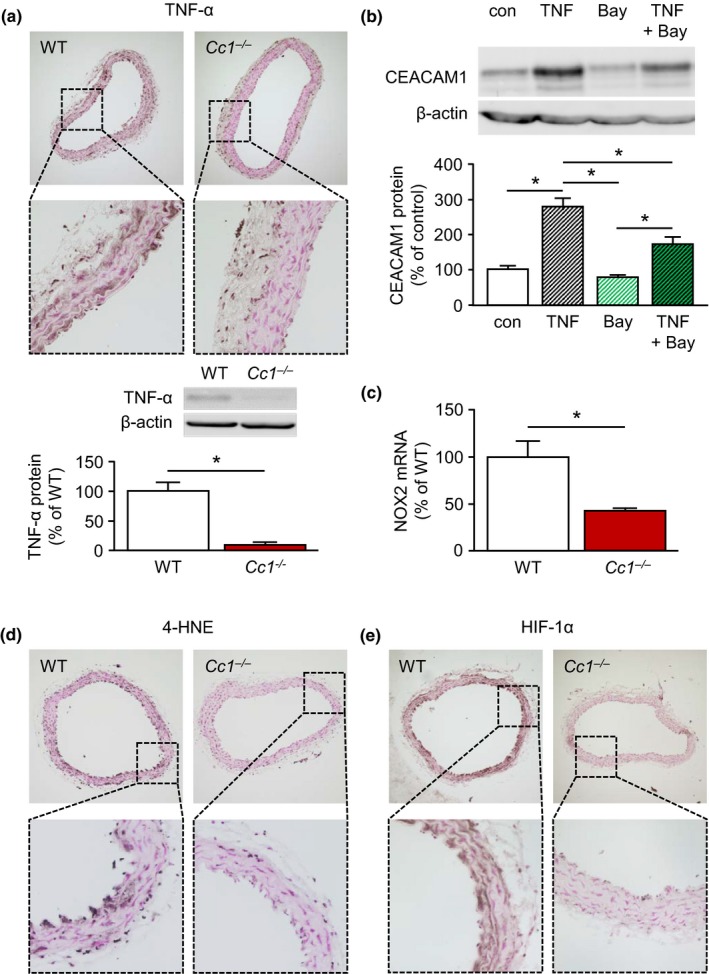
Carcinoembryonic antigen‐related cell adhesion molecule 1 (CEACAM1) promotes age‐dependent upregulation of TNF‐α and oxidative stress. (a) Immunohistochemical analyses of aortic cross sections and immunoblotting demonstrated that CEACAM1 promotes age‐dependent upregulation of TNF‐α in 9‐month‐old mice. (b) Vice versa, TNF‐α (50 ng/ml, 72 hr) upregulated expression of CEACAM1 in cultured endothelial EA.hy926 cells. This TNF‐α‐mediated upregulation of CEACAM1 was partially attenuated by concomitant application of the selective NF‐κB inhibitor Bay 11‐7085 (10 μM, 72 hr). (c) Aortic mRNA expression of the pro‐oxidative NADPH oxidase subunit NOX2 was significantly higher in 9‐month‐old WT mice when compared to age‐matched *Cc1* ^*−/−*^ mice. (d) 4‐HNE, a marker of oxidative stress‐mediated lipid peroxidation accumulated in 9‐month‐old WT mice, but not in *Cc1* ^*−/−*^ mice, as demonstrated by immunohistochemical analyses of aortic cross sections. (e) Compared to *Cc1* ^*−/−*^ mice, the presence of the transcription factor HIF‐1α was increased in aortae of 9‐month‐old WT mice. *p < 0.05

TNF‐α expression in the vasculature increases with progressive age in humans and rodents resulting in increasing vascular oxidative stress (Csiszar et al., 2003; Paolisso et al., 1998; Spaulding et al., 1997; Zhang et al., 2009). In aortae of 9‐month‐old WT mice, we found a higher number of TNF‐α(+) cells and a higher TNF‐α expression compared to aortae of *Cc1* ^*−/−*^ mice (Figure 4a). Vice versa, analysis of human endothelial EA.hy926 cells demonstrated an induction of CEACAM1 expression upon stimulation with TNF‐α, which was partially inhibited by concomitant application of the NF‐κB inhibitor Bay 11‐7085 (Figure 4b).

TNF‐α is known to promote oxidative stress by upregulation of pro‐oxidative enzymes. Accordingly, we found a higher aortic expression of the NADPH oxidase subunit NOX2 in aortae of WT mice compared to *Cc1* ^*−/−*^ mice (Figure 4c). 4‐Hydroxy‐2,3‐trans‐nonenal (4‐HNE), a lipid peroxidation product, is frequently used as a stable marker of oxidative stress (Csala et al., 2015). In 9‐month‐old WT mice, we could demonstrate vascular accumulation of 4‐HNE compared to *Cc1* ^*−/−*^ mice (Figure 4d).

Oxidative stress is known to stabilize HIF‐1α even under normoxic conditions (Kim & Byzova, 2014; Sadaghianloo et al., 2017; Zhou et al., 2007). Accordingly, HIF‐1α protein was detectable in higher amounts in aortae of 9‐month‐old WT mice compared to age‐matched *Cc1* ^*−/−*^ mice, whereas mRNA expression was similar indicating protein stabilization (Figure 4e; Figure S2). Increased presence of TNF‐α, 4‐HNE, and HIF‐1α in the WT was predominantly confined to the intimal and adventitial aortic layers.

### 2.6 Aging‐related upregulation of vascular CEACAM1 expression results in endothelial barrier dysfunction and promotes collagen accumulation

**Figure 5:**
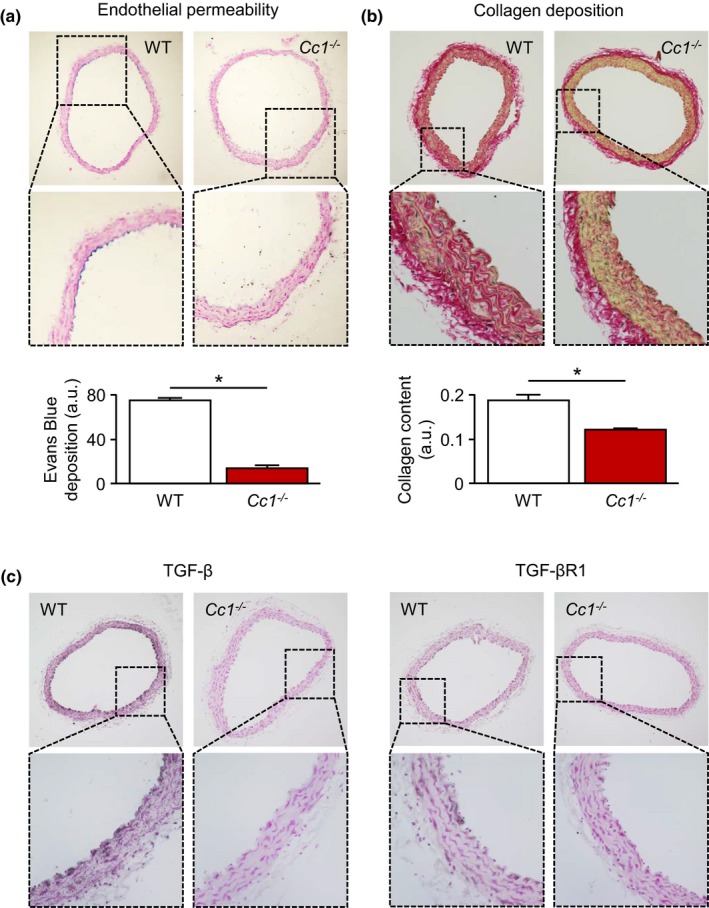
Carcinoembryonic antigen‐related cell adhesion molecule 1 (CEACAM1) promotes age‐dependent vascular hyperpermeability and aortic collagen accumulation. (a) Aortic cross sections of 9‐month‐old WT and *Cc1* ^*−/−*^ mice after in situ perfusion with an Evans Blue‐containing solution. Aortic subendothelial dye deposition was higher in WT mice indicating increased endothelial permeability compared to *Cc1* ^*−/−*^ mice. (b) Detection of collagen by picrosirius red staining of aortic cross sections from 9‐month‐old WT and *Cc1* ^*−/−*^ mice. In the presence of CEACAM1, there was a distinct age‐related accumulation of collagen fibrils, especially within the aortic media. (c) Immunohistochemical analyses of the expression of TGF‐β and TGF‐βR1 in aortic cross sections of 9‐month‐old WT and *Cc1* ^*−/−*^ mice. In WT mice, both proteins were expressed higher suggesting augmented pro‐fibrotic TGF‐β/TGF‐βR1 signaling in the presence of CEACAM1. *p < 0.05

Progressive age is characterized by ED, which includes enhanced endothelial permeability (Bömmel et al., 2017; Ichiki et al., 2013; Phinikaridou et al., 2012; Rodríguez‐Mañas et al., 2009). Therefore, we conducted experiments to evaluate endothelial permeability in WT and *Cc1* ^*−/−*^ mice in situ. Perfusion of the murine circulation with an Evans Blue‐containing solution resulted in intimal dye deposition that is indicative of the endothelial permeability (Bömmel et al., 2017). Intimal Evans Blue deposition was higher in aortae of 9‐month‐old WT mice compared to *Cc1* ^*−/−*^ mice (Figure 5a).

Another hallmark of vascular aging is accumulation of collagen fibrils with subsequent arterial fibrosis (Kohn, Lampi & Reinhart‐King, 2015). Therefore, picrosirius red staining of aortic cross sections was used to determine the aortic collagen content in WT and *Cc1* ^*−/−*^ mice, respectively. These analyses clearly demonstrated higher collagen content, especially within the aortic media of 9‐month‐old WT mice compared to *Cc1* ^*−/−*^ mice (Figure 5b). Intriguingly, the collagen content of aortae from 9‐month‐old *Cc1* ^*−/−*^ mice was comparable to that of 2‐month‐old WT and *Cc1* ^*−/−*^ mice, respectively, indicating a complete lack of age‐associated aortic collagen deposition due to CEACAM1 deficiency (Figure S3). TGF‐β/TGF‐βR1 signaling is known to stimulate collagen expression and deposition (Kim, Sheppard & Chapman, 2018). Accordingly, expression of TGF‐β and TGF‐βR1 was higher in aortae of 9‐month‐old WT mice compared to age‐matched *Cc1* ^*−/−*^ mice (Figure 5c; Figure S4).

### 2.7 Markers of inflammation, oxidative stress, and fibrosis are differentially expressed in 6‐ and 9‐month‐old mice

In aortae of 9‐month‐old mice, we found that markers of inflammation, oxidative stress, and fibrosis are more abundant in WT mice compared to *Cc1* ^*−/−*^ mice. This contrasts with a previous report on the expression of those markers in 6‐month‐old WT and *Cc1* ^*−/−*^ mice (Najjar et al., 2013). In order to verify our genetic mouse model, we re‐examined the presence of TNF‐α, 4‐HNE, and TGF‐β in aortic cross sections from 6‐month‐old WT and *Cc1* ^*−/−*^ mice. In these immunohistochemical analyses, we found that similar to Najjar et al. (2013), all three markers were more abundant in *Cc1* ^*−/−*^ mice (Figure S5).

### 2.8 Aging‐associated vascular effects of CEACAM1 deficiency and TNF‐α are partially mimicked by CEACAM1 knockdown in endothelial cells

**Figure 6:**
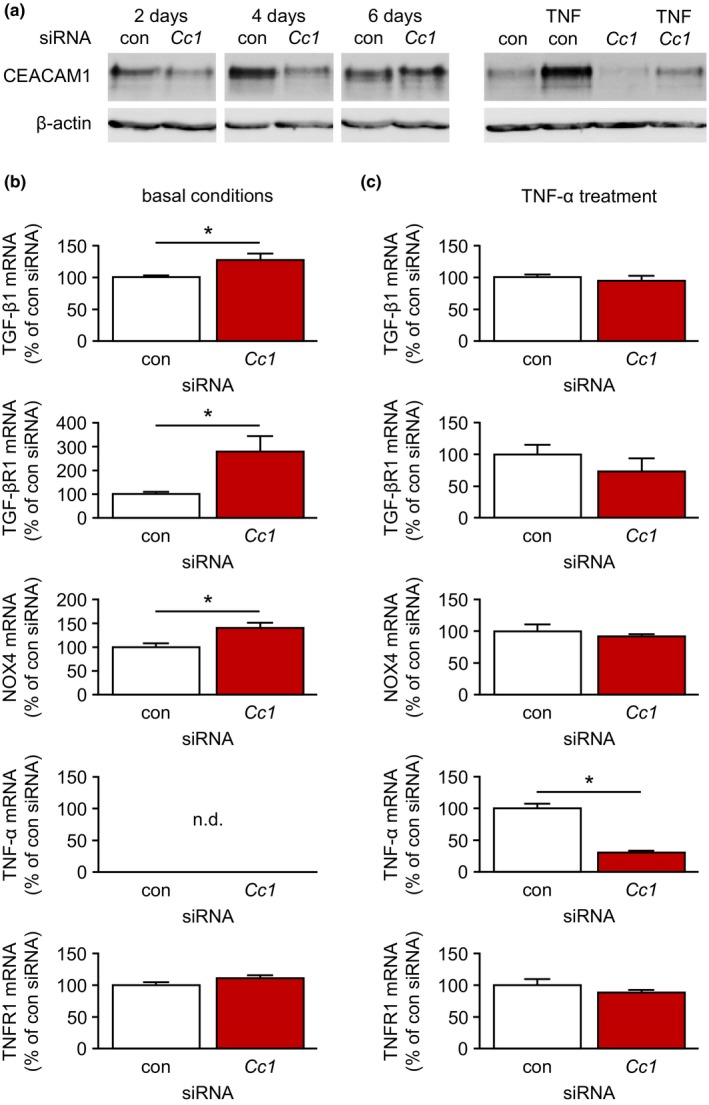
Knockdown of carcinoembryonic antigen‐related cell adhesion molecule 1 (CEACAM1) in endothelial cells in vitro mimics in vivo effects of CEACAM1 deficiency on fibrosis‐, oxidative stress‐, and inflammation‐related genes. (a) Immunoblot detection of CEACAM1 protein in control siRNA or *Cc1* siRNA‐transfected EA.hy926 cells after different time points and upon TNF‐α application. CEACAM1 protein was reduced by approximately 50% 2–4 days after *Cc1* siRNA transfection. TNF‐α (50 ng/ml, 72 hr) induced a huge upregulation of CEACAM1 protein in control siRNA‐transfected cells. However, even in the presence of TNF‐α, *Cc1* siRNA efficiently knocked down CEACAM1 protein. (b) Analysis of mRNA expression under basal conditions 4 days after control and *Cc1* siRNA transfection, respectively. Expression of TGF‐β, TGF‐βR1, and NOX4 was significantly higher due to CEACAM1 knockdown, whereas TNFR1 was not affected. TNF‐α was not detectable (n.d.) under basal conditions. (c) Analysis of mRNA expression 4 days after control and *Cc1* siRNA transfection, respectively. One day after transfection, both groups of cells were stimulated with TNF‐α (50 ng/ml) for the remaining 72 hr. The differences in the expression of TGF‐β, TGF‐βR1, and NOX4 between control and *Cc1* siRNA‐transfected cells seen under basal conditions were equalized by TNF‐α. Again, expression of TNFR1 was unaffected by the treatment. However, TNF‐α induced its own expression in both groups of cells. This induction was much more pronounced in control siRNA‐transfected cells. *p < 0.05

In order to analyze the effect of CEACAM1 on the expression of fibrosis‐, oxidative stress‐, and inflammation‐related genes under basal conditions and upon TNF‐α treatment in vitro, we knocked down CEACAM1 expression in EA.hy926 cells using the siRNA technique. CEACAM1 protein expression was reduced by approximately 50% 2–4 days after transfection of cells with a CEACAM1‐specific siRNA when compared to cells transfected with a CEACAM1‐unrelated control siRNA even in the presence of TNF‐α (Figure 6a). Therefore, we analyzed mRNA expression in EA.hy926 cells 4 days after transfection. Under basal conditions, mRNA expression of TGF‐β, TGF‐βR1, and NOX4 was significantly increased by CEACAM1 knockdown (Figure 6b). TNFR1 was not affected by transfection of CEACAM1‐specific siRNA, and TNF‐α was not detectable under basal conditions (Figure 6b). TNF‐α treatment abolished the differences in the expression of TGF‐β, TGF‐βR1, and NOX4 between control and *Cc1* siRNA‐transfected cells that were present under basal conditions (Figure 6c). Furthermore, TNF‐α induced its own mRNA expression in EA.hy926 cells. This TNF‐α‐induced TNF‐α mRNA expression was much higher in cells transfected with control siRNA (Figure 6c). Finally, mRNA expression of TNFR1 was not different between TNF‐α‐treated control and *Cc1* siRNA‐transfected cells (Figure 6c).

## 3 DISCUSSION

Cardiovascular diseases such as myocardial infarction and stroke are the leading cause of death worldwide (Gisterå & Hansson, 2017). In the last decades, cardiovascular research focused on pathological alterations, that is, atherosclerotic plaque formation using genetic mouse models (Gisterå & Hansson, 2017; Lee et al., 2016; Whitman, 2004). In contrast, the influence of physiological aging on the vasculature and the triggers that cause transition into pathological conditions are less well examined (Wang & Bennett, 2012). However, understanding vascular aging processes and the transition into angiopathies is of great significance in order to develop therapeutic strategies to prevent myocardial infarction and stroke.

Carcinoembryonic antigen‐related cell adhesion molecule 1 is known to play a crucial role in the homeostasis of blood vessels (Ghavampour et al., 2018; Najjar et al., 2013). In the present study, we prove for the first time a role of CEACAM1 in vascular aging processes in mice and humans. In brief, we show that: (a) HITA represents a suitable model to investigate physiological vascular aging; (b) with increasing age, TNF‐α induces CEACAM1 expression, which in turn upregulates TNF‐α, thus maintaining a pro‐inflammatory milieu; (c) this results in enhanced oxidative stress stabilizing HIF‐1α; (d) HIF‐1α contributes to endothelial barrier impairment via VEGF/VEGFR‐2 signaling; and (e) CEACAM1 contributes to age‐dependent vascular fibrosis via augmented TGF‐β/TGF‐βR1 signaling.

Investigation of physiological vascular aging in humans is difficult due to limited availability of human tissue samples (Wang et al., 2007). In the present study, we made use of HITA specimens for two reasons. First, HITA is the preferred vessel for human coronary bypass graft surgery due to its superior long‐term patency compared to other vessel grafts (King, 2011; Otsuka, Yahagi, Sakakura & Virmani, 2013). Therefore, surplus fragments of HITA that are cut during aortocoronary bypass surgery are accessible for further analyses. Second, in order to study physiological age‐associated processes, the samples should be as free as possible from pathological alterations. HITA is well known for its resistance against atherosclerotic plaque formation (Singh, 1983). In our study, we confirmed this morphological stability of HITA in comparison with radial artery specimens. HITA specimens displayed almost no pathological alterations such as inflammatory cell accumulation, intimal thickening, or atherosclerotic plaque formation compared to radial arteries, even at advanced age. These findings further support the suitability of HITA to study physiological vascular aging in humans.

Vascular aging is accompanied by the development of ED (Rodríguez‐Mañas et al., 2009), which is, among others, characterized by enhanced vascular permeability (Bömmel et al., 2017; Ichiki et al., 2013; Phinikaridou et al., 2012). Recently, we showed that CEACAM1 contributes to an increased endothelial permeability in 9‐month‐old mice (Ghavampour et al., 2018). Therefore, we wondered whether CEACAM1 expression is age‐dependently regulated. Analyzing HITA specimens from patients of different age, we found that expression of CEACAM1 is higher in older donors. Interestingly, expression of VEGF and the transcription factor HIF‐1α that mediates VEGF transcription in response to hypoxia were similarly upregulated in HITA from older donors. VEGF is a potent mediator of endothelial permeability (Bates, 2010) that is induced by CEACAM1 overexpression (Kilic et al., 2005). Therefore, it is feasible to speculate that CEACAM1 is responsible for the vascular upregulation of VEGF in older donors. Unfortunately, a causal relationship between CEACAM1 and VEGF upregulation cannot be tested using the HITA model.

To answer that question, we used a murine model of genetic ablation of CEACAM1, the *Cc1* ^*−/−*^ mouse (Leung et al., 2006; Najjar et al., 2013). We analyzed 2‐month‐old mice compared to 9‐month‐old mice. At 9 months, mice represent middle‐aged mature adults (Fox et al., 2007), a phase of life known to be associated with first pathological vascular alterations in humans (Tuzcu et al., 2001). First, we verified the suitability of the murine model. Similar to HITA, we detected an upregulation of CEACAM1 and VEGF in the aortae of the older group. Additionally, aortic VEGFR‐2 expression was higher in 9‐month‐old mice, further supporting the assumption of augmented vascular VEGF signaling with increasing age.

The first hint for an involvement of CEACAM1 in age‐associated increased VEGF signaling came from co‐immunohistochemical analyses of aortae from 9‐month‐old WT mice. In these samples, an extensive co‐localization of CEACAM1 with VEGF and VEGFR‐2, respectively, was detected. More importantly, we found that the number of VEGF(+) and VEGFR‐2(+) cells, as well as protein expression of VEGF and VEGFR‐2, was tremendously reduced in aortae of 9‐month‐old *Cc1* ^*−/−*^ mice. This clearly proves a crucial role of CEACAM1 for the age‐dependent upregulation of VEGF and VEGFR‐2 expression and confirms to a previous report showing CEACAM1‐mediated VEGF expression in endothelial cells (Kilic et al., 2005). Since VEGF/VEGFR‐2 signaling is one of the most potent inducers of vascular hyperpermeability (Bates, 2010), it is not surprising that CEACAM1 also affects endothelial permeability in 9‐month‐old mice. Compared to *Cc1* ^*−/−*^ mice, intimal Evans Blue deposition as a marker of endothelial permeability was much higher in aortae from WT mice.

Next, we aimed to elucidate the mechanism that was responsible for CEACAM1‐dependent aortic VEGF/VEGFR‐2 upregulation and increased vascular permeability in 9‐month‐old WT mice. Vascular expression and circulating levels of TNF‐α, a potent pro‐inflammatory peptide, are increased in aging rodents and humans (Csiszar et al., 2003; Paolisso et al., 1998; Spaulding et al., 1997). Therefore, it represents an attractive candidate for mediating age‐dependent vascular alterations, that is, via increased oxidative stress (Chen et al., 2008; Rodríguez‐Mañas et al., 2009). Using our murine model, we demonstrated that the vascular expression of TNF‐α in 9‐month‐old mice critically depends on the presence of CEACAM1. This conclusion is further supported by our finding that the TNF‐α‐induced TNF‐α expression is impaired in endothelial cells with CEACAM1‐specific knockdown. Moreover, we showed that TNF‐α robustly induces the expression of CEACAM1 in cultured human endothelial cells, an effect partially mediated by NF‐κB activation. Based on these data, we assume that in the aging vasculature, a vicious cycle is established by mutual upregulation of CEACAM1 and TNF‐α expression that contributes to the maintenance of a pro‐inflammatory milieu (Gisterå & Hansson, 2017; Wu, Li, Hou, & Chu, 2017).

TNF‐α was shown to promote formation of reactive oxygen species, that is, by upregulation of vascular NADPH oxidases (Moe et al., 2006; Muzaffar, Shukla, Angelini & Jeremy, 2004). Accordingly, in aortae of 9‐month‐old WT mice expression of the NADPH oxidase subunit NOX2 was higher compared to *Cc1* ^*−/−*^ mice. Additionally, 4‐HNE, a stable lipid peroxidation product indicative of oxidative stress, was more abundant in aortae of WT mice. Increased oxidative stress in 9‐month‐old WT mice is of special interest, because it was shown that oxidative stress stabilizes HIF‐1α even under normoxic conditions (Kim & Byzova, 2014; Sadaghianloo et al., 2017; Zhou et al., 2007) and HIF‐1α mediates VEGF gene transcription (Shibuya, 2011). Indeed, the presence of HIF‐1α protein was higher in aortae of 9‐month‐old WT mice compared to *Cc1* ^*−/−*^ mice, whereas mRNA expression was not different. This supports the conclusion of a stabilization of HIF‐1α protein in 9‐month‐old WT mice. Since HIF‐1α activity was shown to promote also NOX2 expression (Diebold et al., 2012), the establishment of a vicious cycle augmenting vascular inflammation in aged vessels can be assumed. Summarizing these data, we found evidence that in the aging murine aorta, CEACAM1 contributes to an upregulation of TNF‐α, which stimulates VEGF/VEGFR‐2 signaling via oxidative stress‐mediated HIF‐1α stabilization, thereby promoting increased endothelial permeability. In addition, we showed previously that on the cellular and organ level, TNF‐α increases endothelial permeability in the presence of CEACAM1, but reduces it in the absence of CEACAM1 (Ghavampour et al., 2018). This differential TNF‐α‐mediated effect could also contribute to the increased permeability in 9‐month‐old WT mice compared to age‐matched *Cc1* ^*−/−*^ mice.

Increased endothelial permeability is characteristic of age‐associated ED. Beside these functional alterations, fragmentation of elastic fibers and deposition of less compliant collagen fibers leading to vascular fibrosis accompany vascular aging (Wang & Bennett, 2012). Those structural age‐dependent alterations seem to depend on CEACAM1, too. Whereas in 9‐month‐old WT mice, a tremendous deposition of collagen, especially within the aortic media is detectable, aortic media of age‐matched *Cc1* ^*−/−*^ mice are almost free of collagen. Thus, concerning collagen deposition, aortae of 9‐month‐old *Cc1* ^*−/−*^ mice are indistinguishable from those of 2‐month‐old WT and *Cc1* ^*−/−*^ mice, respectively. TGF‐β/TGF‐βR1 signaling is an important factor promoting tissue collagen deposition (Kim et al., 2018). Since expression of TGF‐β and TGF‐βR1 is significantly lower in aortae of 9‐month‐old *Cc1* ^*−/−*^ mice compared to WT mice, reduced TGF‐β/TGF‐βR1 signaling may be responsible for the lack of age‐associated collagen deposition in CEACAM1 deficiency.

In an attempt to strengthen the conclusions drawn from the in vivo data, we established an endothelial cell culture model. Transfection of EA.hy926 cells with CEACAM1‐specific siRNA reduced CEACAM1 protein expression by approximately 50%. The reduced abundance of CEACAM1 resulted in an increased expression of the pro‐fibrotic genes TGF‐β and TGF‐βR1. Furthermore, the NADPH oxidase subunit NOX4 was upregulated. At first glance, these data seem to contradict our results from 9‐month‐old mice showing a reduced presence of TGF‐β, TGF‐βR1, and oxidative stress marker within the aortic wall due to CEACAM1 deficiency. However, these data conform to results of a study on 6‐month‐old WT and *Cc1* ^*−/−*^ mice published by Najjar et al. (2013). Upregulation of inflammatory and pro‐oxidative markers in 6‐month‐old *Cc1* ^*−/−*^ mice was ascribed to hyperinsulinemia due to reduced hepatic insulin clearance (Najjar et al., 2013). However, our results obtained from cultured EA.hy926 support a rather endothelial cell‐autonomous effect of CEACAM1 deficiency on the expression of TGF‐β1 and NOX4. With respect to TNF‐α, systemic factors, that is, hyperinsulinemia, might trigger the increased arterial presence of this pro‐inflammatory cytokine in 6‐month‐old *Cc1* ^*−/−*^ mice as suggested, since we were unable to detect TNF‐α in our cultured endothelial cells under basal conditions. This demonstrates that under basal conditions, our cell culture model reproduces the situation in WT and *Cc1* ^*−/−*^ mice at a younger stage.

With advancing age, circulating levels as well as vascular expression of TNF‐α increase (Csiszar et al., 2003; Paolisso et al., 1998; Spaulding et al., 1997). The actual trigger of this process is unknown. We aimed to mimic this situation in vitro by TNF‐α treatment of control and *Cc1* siRNA‐transfected EA.hy926 cells. Upon treatment with TNF‐α, the CEACAM1‐mediated differences in the expression of TGF‐β, TGF‐βR1, and NOX4 detected under basal conditions were equalized. Hence, TNF‐α application induced a trend toward the aortic expression ratios detected in 9‐month‐old WT and *Cc1* ^*−/−*^ mice. In vivo, the effect of TNF‐α might be much more pronounced in WT mice, because our endothelial cell culture experiments demonstrate an upregulation of CEACAM1 expression by TNF‐α and, in turn, an augmented TNF‐α‐induced endothelial TNF‐α expression in the presence of CEACAM1. Therefore, these data suggest an amplification of TNF‐α‐mediated age‐related effects that takes place in WT but is absent in *Cc1* ^*−/−*^ mice resulting in a switch in the vascular expression profile of WT and *Cc1* ^*−/−*^ mice between 6 and 9 months of age.

**Figure 7:**
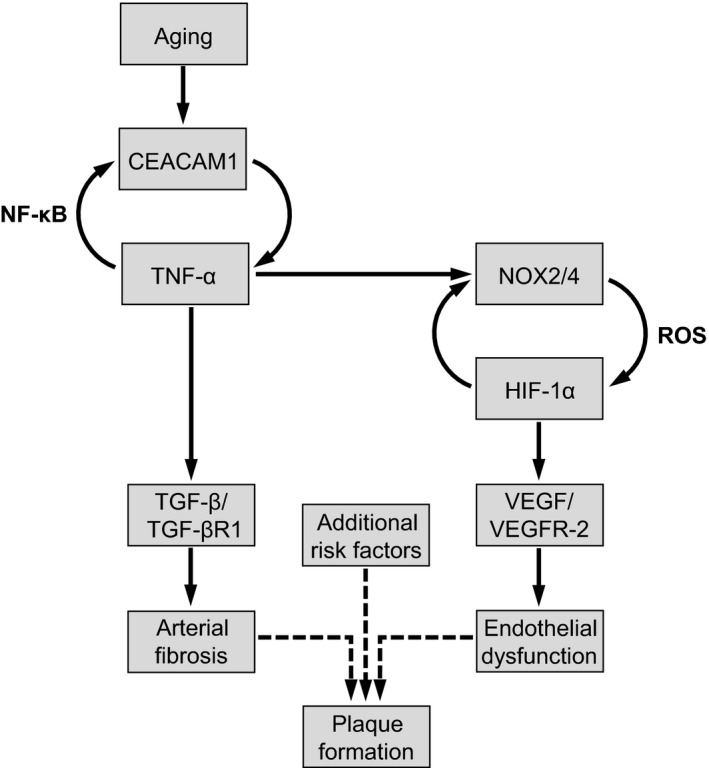
Carcinoembryonic antigen‐related cell adhesion molecule 1 (CEACAM1) as a central mediator of vascular aging. In the course of vascular aging, CEACAM1 expression is increased. CEACAM1 promotes aortic upregulation of the pro‐inflammatory cytokine TNF‐α that, in turn, further contributes to an enhanced CEACAM1 expression. This mutual influence establishes a feed‐forward loop maintaining a chronic pro‐inflammatory milieu within the vessel wall. Upregulated TNF‐α may induce expression of pro‐oxidative enzymes, that is, NOX2, that contribute to an oxidative stress‐mediated stabilization of HIF‐1α. HIF‐1α that induces VEGF/VEGFR‐2 signaling thus promotes endothelial hyperpermeability of aged vessels. Beside this pathway leading to age‐related endothelial dysfunction, upregulated TNF‐α may also account for the age‐dependently increased arterial fibrosis via enhanced TGF‐β/TGF‐βR1 signaling. In the presence of additional risk factors, these CEACAM1‐mediated age‐dependent vascular alterations may transform into the development of vascular disorders, that is, atherosclerosis

In summary, we clearly show that CEACAM1 contributes to different structural and functional aspects of vascular aging. Some of these CEACAM1‐dependent effects, that is, increased oxidative stress, increased VEGF/VEGFR‐2 signaling, and increased endothelial permeability, may render aged blood vessels prone to the onset of vascular disorders such as atherosclerosis in the presence of additional risk factors (Figure 7). Therefore, CEACAM1 function may represent a potential target in order to delay unfavorable age‐dependent vascular alterations, thereby preventing the onset of transition from physiological aging into angiopathies with their secondary diseases such as myocardial infarction and stroke.

## 4 EXPERIMENTAL PROCEDURES

### 4.1 Animals

*Cc1* ^*−/−*^ mice (Leung et al., 2006; Najjar et al., 2013) and their WT littermates (C57BL/6J; The Jackson Laboratory) were housed in specific pathogen‐free conditions on a 12:12‐hour dark–light cycle and fed standard chow ad libitum. Mice were analyzed at the age of 2 and 9 months, respectively. All animal experiments were approved by local authorities (Regierung von Unterfranken) to comply with German animal protection law.

### 4.2 Human tissue samples

Human internal thoracic artery fragments were obtained during aortocoronary bypass surgery in the Clinics of Thoracic and Cardiovascular Surgery, University Hospital Essen, without additional burden for the patients according to local ethical and biohazard regulations. All studies analyzing human tissue samples were approved by the local ethics committee. Informed consent form (Nr. 10‐4363) was provided by the ethics commission of the University Medical Faculty Essen. HITA fragments were grouped according to patients’ age into three groups: <60, 60–70, and >70 years based on preliminary results.

### 4.3 Cell culture

Endothelial EA.hy926 cells (ATCC® CRL‐2922™; ATCC, LGC Standards GmbH) were cultured in DMEM/10% FCS (Thermo Fisher; Biochrom GmbH) at 37°C/5% CO_2_. For stimulation experiments, cells were incubated with 50 ng/ml TNF‐α (Thermo Fisher) for 72 hr. Bay 11‐7085 (Cayman Chemical; 10 μM) was used to inhibit NF‐κB activation.

### 4.4 CEACAM1 knockdown

Carcinoembryonic antigen‐related cell adhesion molecule 1 protein expression was knocked down in endothelial EA.hy926 cells using a siRNA specific for human *Cc1* (CEACAM1 siRNA (h); Santa Cruz Biotechnology). An unrelated siRNA served as control (Control siRNA‐A; Santa Cruz Biotechnology). Transfection of cells with siRNAs was carried out using siRNA Transfection Reagent and siRNA Transfection Medium according to the manufacturer's instructions (8 μl scale; Santa Cruz Biotechnology). The knockdown effect was monitored up to 6 days. CEACAM1 protein expression was reduced by approximately 50% 2–4 days after transfection of cells.

### 4.5 Immunohistochemistry, immunoblotting, and cell count analysis

Immunohistochemical analyses were conducted as described previously (Ergün et al., 2004; Ghavampour et al., 2018). The immunoreaction was detected using peroxidase–antiperoxidase and avidin–biotin complex methods by a modified nickel‐enhanced glucose oxidase technique (Kilic & Ergün, 2001). Immunoblot experiments were conducted according to a protocol described before (Ghavampour et al., 2018; Oliveira‐Ferrer et al., 2004). Primary antibodies used in this study are listed in Table S2. Secondary antibodies used for immunohistochemical or immunoblot analyses were provided by Dako (Agilent) and Pierce (Thermo Fisher Scientific), respectively.

Cell counts of immunostained intimal and medial cells and endothelial circumference were determined using ImageJ software (Wayne Rasband, NIH1.46r) as described before (Jensen, 2013). Cell counts of immunostained cells were normalized to the endothelial circumference of the respective aortic cross section.

### 4.6 Picrosirius red staining

Picrosirius red staining was performed according to a protocol described previously (Vogel, Siebert, Hofmann & Frantz, 2015). After de‐waxing and hydration, paraffin‐embedded tissue sections were stained with Weigert's hematoxylin. Collagen fibers within the tissue samples were stained for 1 hr using 1% Direct Red 80 in saturated aqueous solution of picric acid (both from Sigma‐Aldrich). Tissue sections were washed twice with acidified water and were dehydrated using ethanol solutions. ImageJ software (Wayne Rasband, NIH1.46r) was used to quantify the collagen content that was normalized to the endothelial circumference.

### 4.7 Permeability assay

Vascular permeability assays were performed as described previously (Bömmel et al., 2017). Briefly, after cervical dislocation blood was removed by flushing the circulation with Ringer solution through the left ventricle. Thereafter, vasculature of mice was perfused with the dye Evans Blue (0.03% in Ringer solution, color index number: 23,860; Sigma‐Aldrich). Depending on the vascular permeability, Evans Blue was able to diffuse into the subendothelial compartment of blood vessels. After 30 min, the remaining intravascular dye was washed away using PBS. Aortae were freed from perivascular fat, dissected out, and fixed with 4% PFA for 1 hr. After washing with PBS, the thoracic aorta was frozen in Tissue‐Tek^®^ (Sakura Finetek Europe B.W., NL). Ten micrometer cross‐cryosections were prepared and subsequently counterstained with calcium red (Sigma‐Aldrich). Images were taken from at least 10 randomly selected sections using a Keyence BZ9000 microscope (Keyence). ImageJ software (Wayne Rasband, NIH1.46r) was used to determine the portion of the intima that was stained by Evans Blue in these sections. The dye‐stained length normalized to the total intimal circumference was used as a marker of endothelial permeability.

### 4.8 Quantitative real‐time PCR

After isolation of total RNA (Qiazol; Qiagen Sciences Inc) and DNase I treatment (Promega), RNA was transcribed into cDNA (SuperScript II; Thermo Fisher Scientific). For each sample, mRNA expression was analyzed by real‐time PCR in triplicate (GoTaq qPCR Master Mix; Promega). Carryover of genomic DNA was monitored by running an amplification with SuperScript II‐deficient RT reaction as template for each sample. Primers used in this study (Eurofins Genomics) are listed in Table S3. After completion of the PCR cycling protocol (95°C for 30 s; 60°C for 30 s; 72°C for 30 s; 40×), specificity of amplification was proven by melting curve analyses. Values for the mRNA of the respective genes were normalized to glyceraldehyde‐3‐phosphate dehydrogenase mRNA and are given as arbitrary units (a.u.) or as percent of control siRNA.

### 4.9 Statistical analyses

All experiments were repeated at least three times with similar results. Data are presented as mean ± sem and were compared using two‐tailed Student's *t* test or analysis of variance (ANOVA) when appropriate. The null hypothesis was rejected at *p *<* *.05 (*).*p *<* *.001 was considered highly significant (***).

## CONFLICT OF INTEREST

None declared.

## AUTHOR CONTRIBUTIONS

F. Kleefeldt, U. Rueckschloss, and S. Ergün were responsible for study design; F. Kleefeldt, H. Bömmel, B. Broede, M. Thomsen, V. Pfeiffer, P. Wörsdörfer, N. Wagner, S. Karnati, and U. Rueckschloss conducted the experimental work; F. Kleefeldt, U. Rueckschloss, and S. Ergün performed the data analysis; F. Kleefeldt, H. Bömmel, U. Rueckschloss, and S. Ergün were responsible for manuscript writing and review; and S. Ergün was responsible for grant acquisition and general supervision.

## Supporting information

D Click here for additional data file.

D Click here for additional data file.
