# Supplementary material for: Aging‐related carcinoembryonic antigen‐related cell adhesion molecule 1 signaling promotes vascular dysfunction
Source: Aging Cell. 2019 Aug 6;18(6):e13025. doi: 10.1111/acel.13025 (PMC6826129; doi:10.1111/acel.13025)

Suppl.Fig.1

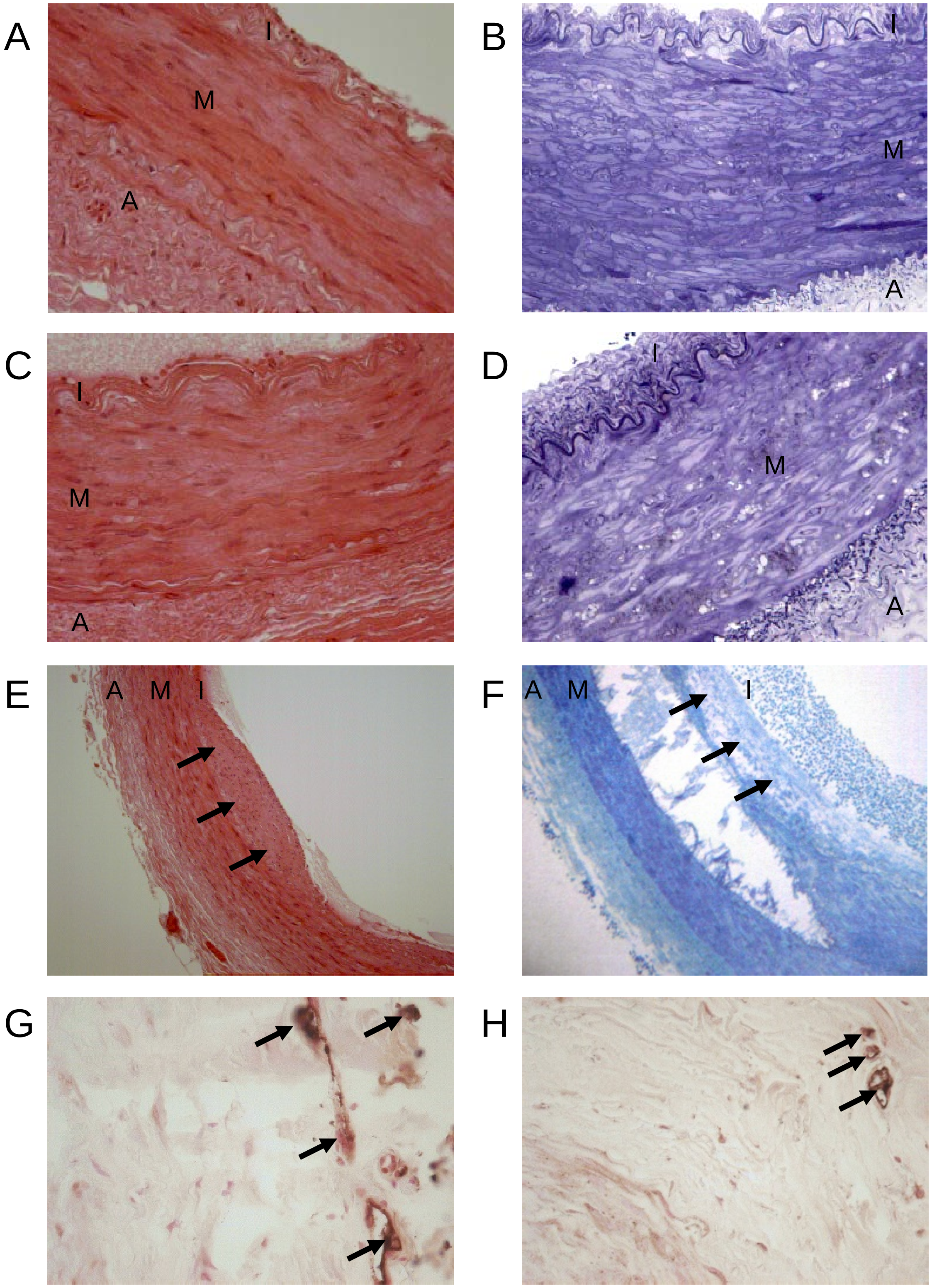

Suppl.Fig.2

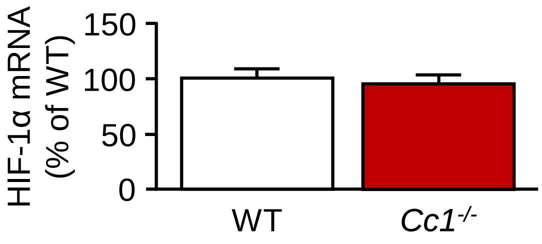

Suppl.Fig.3

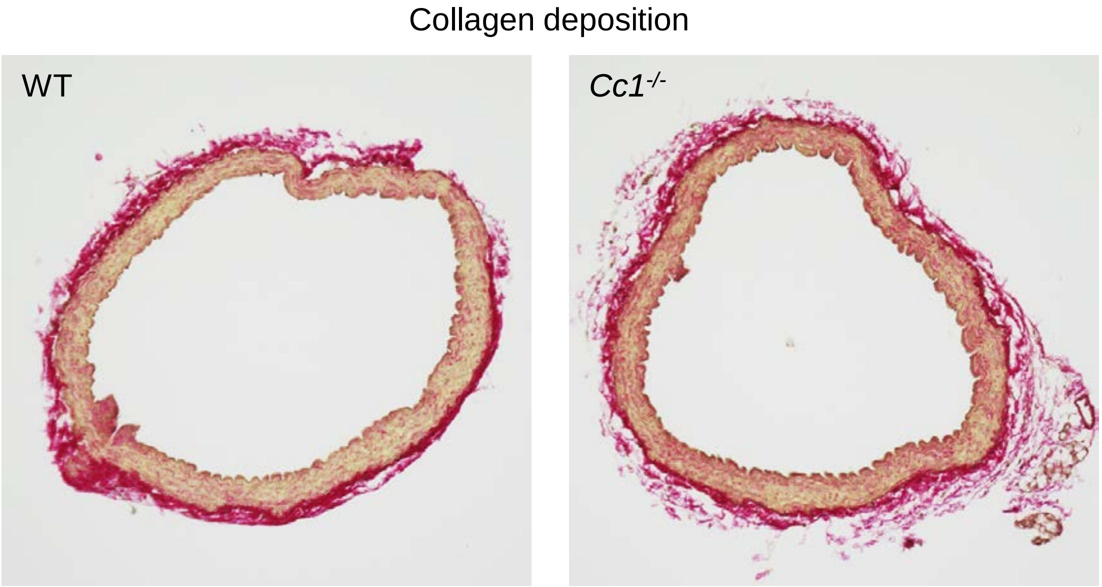

Suppl.Fig.4

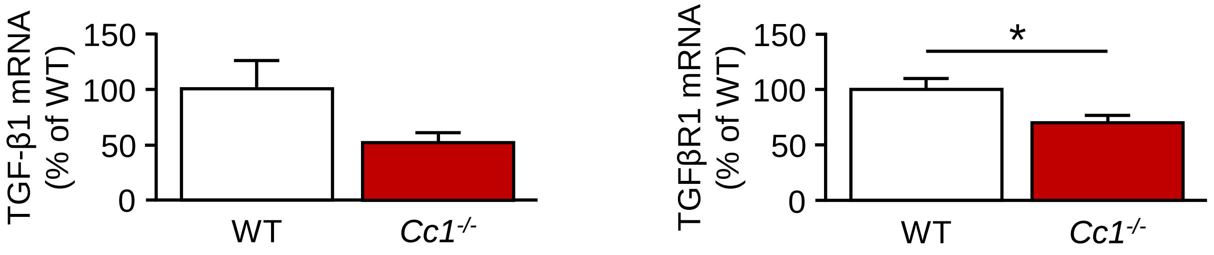
TGF-β1 mRNA
(% of WT)
150
100
50
0
WT
Cc1-/-
TGFβR1 mRNA
(% of WT)
150
100
50
0
*
WT
Cc1-/-

Suppl.Fig.5

WT | *Cc1*$^{-/-}$

TNF-α

4-HNE

TGF-β

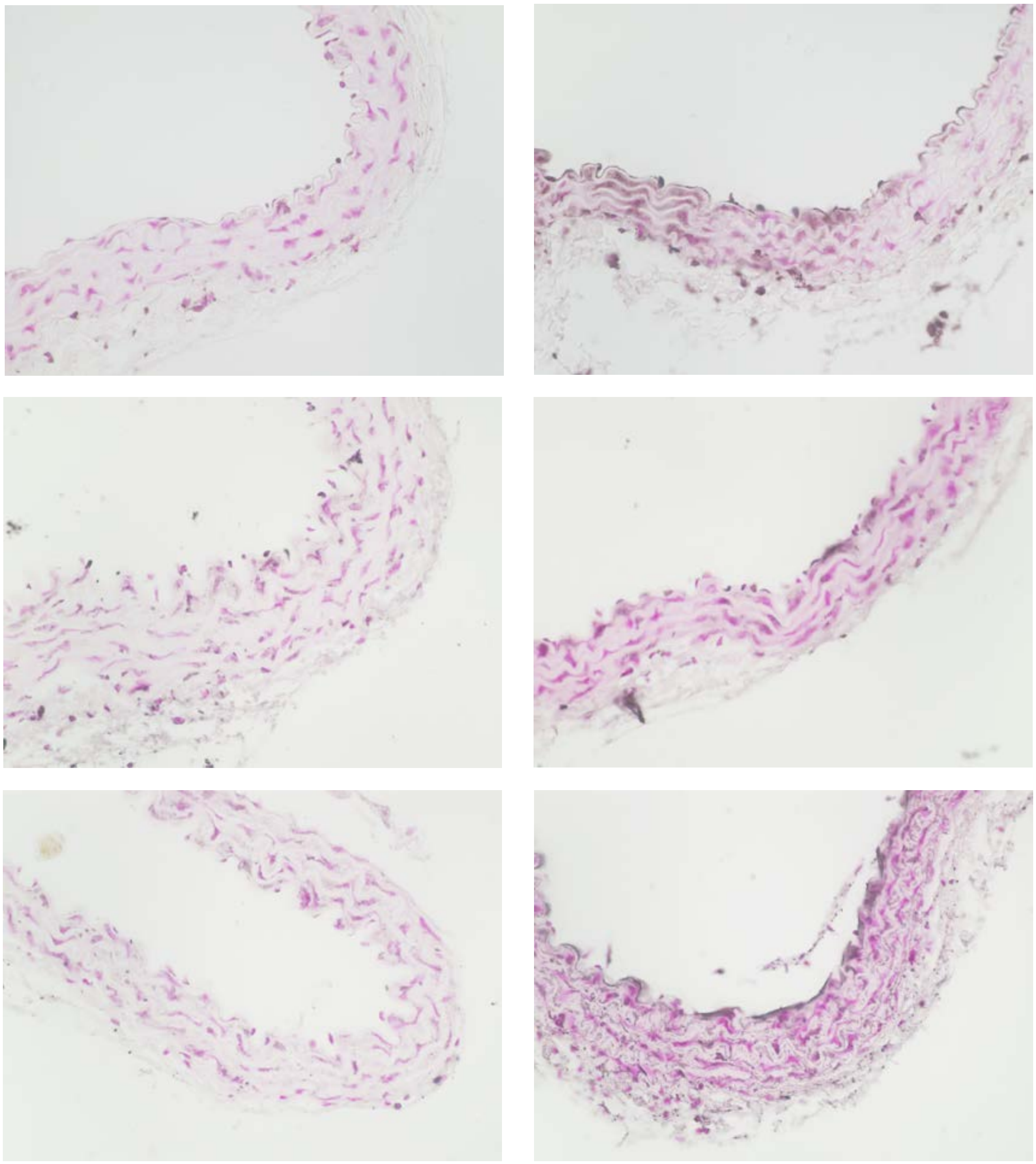

Supplement: Supplementary file 1 — D [file ACEL-18-e13025-s001.pdf]
