# Supplementary material for: Aging‐related carcinoembryonic antigen‐related cell adhesion molecule 1 signaling promotes vascular dysfunction
Source: Aging Cell. 2019 Aug 6;18(6):e13025. doi: 10.1111/acel.13025 (PMC6826129; doi:10.1111/acel.13025)
Supplement: Supplementary file 2 — D [file ACEL-18-e13025-s002.docx]

**Supporting Information listing**

- Suppl.Fig.1
- Suppl.Fig.2
- Suppl.Fig.3
- Suppl.Fig.4
- Suppl.Fig.5
- Suppl.Tab.1
- Suppl.Tab.2
- Suppl.Tab.3

**Tables**

**Suppl.Tab.1:** Characterization of donors of HITA specimens used in this study.

| Risk factors | < 60 years  n = 34 | 60-70 years  n = 33 | > 70 years  n = 39 |
| --- | --- | --- | --- |
| Arterial Hypertension | 21 | 29 | 39 |
| Hyperlipoproteinemia | 22 | 27 | 25 |
| Diabetes mellitus | 7 | 4 | 11 |
| Smoking | 19 | 5 | 2 |
| Positive family history of cardiovascular events | 6 | 11 | 9 |
| General vascular sclerosis | 3 | 6 | 7 |

**Suppl.Tab.2:** Antibodies used in this study.

| Protein | Supplier | Order No. |
| --- | --- | --- |
| CEACAM1 (4D1C2) | Novocastra Laboratories | - |
| CEACAM1 (mCc1) | Kathryn Holmes - University of Colorado, Denver, CO, USA | - |
| HIF-1α | Santa Cruz | sc-13515 |
| 4-HNE | Bioss | bs-6313R |
| TGF-β | St John’s Laboratory | STJ95997 |
| TGFβR1 | St John’s Laboratory | STJ95992 |
| TNF-α | Santa Cruz | sc-52746 |
| VEGF-A | Abcam | ab46154 |
| VEGFR-2 (Flk-1) | Santa Cruz | sc-48161 |

**Suppl.Tab.3:** Real-time PCR primers used in this study.

| Gene | Gene bank Acc. No. | Upstream Primer  (5’ → 3’) | Downstream Primer  (5’ → 3’) |
| --- | --- | --- | --- |
| murine |  |  |  |
| NOX2 | NM_007807.5 | TATGCTGATCCTGCTGCCAGT | TGTCTTCGAATCCTTGTCGAGC |
| TGF-β1 | NM_011577.2 | CCTTCCTGCTCCTCATGG | CGCACACAGCAGTTCTTCTC |
| TGFβR1 | NM_009370.3 | AGAAGAGCGTTCATGGTTCC | CGTCCATGTCCCATTGTCT |
| HIF-1α | NM_010431.2 | CTCAGTCGACACAGCCTCGAT | CTGTGGCTGGGAGTTCTTCGT |
| human |  |  |  |
| NOX4 | NM_001143837.1 | TCCAGTCCTTCCGTTGGTTTGC | TTGGGTCCACAACAGAAAACACCA |
| TGF-β1 | NM_000660.6 | AGAAGCGGTACCTGAACCCG | GCCGGTAGTGAACCCGTTGA |
| TGFβR1 | NM_004612.4 | GTTCGTGGTTCCGTGAGGCA | AAGATGGGCAAGACCGCTCG |
| TNF-α | NM_000594.4 | AGGCGGTGCTTGTTCCTCAG | ACAGGCTTGTCACTCGGGGT |
| TNFR1 | NM_001346091.1 | CAATGGGACCGTGCACCTCT | GGCACAACTTCGTGCACTCC |
